# An updated review of mechanistic potentials of melatonin against cancer: pivotal roles in angiogenesis, apoptosis, autophagy, endoplasmic reticulum stress and oxidative stress

**DOI:** 10.1186/s12935-021-01892-1

**Published:** 2021-03-31

**Authors:** Saeed Mehrzadi, Mohammad Hossein Pourhanifeh, Alireza Mirzaei, Farid Moradian, Azam Hosseinzadeh

**Affiliations:** 1 Razi Drug Research Center, Iran University of Medical Sciences, Tehran, Iran; 2 Bone and Joint Reconstruction Research Center, Shafa Orthopedic Hospital, Iran University of Medical Sciences, Tehran, Iran; 3 Cancer Research Center, Shahid Beheshti University of Medical Sciences, Tehran, Iran; 4 Research Center for Biochemistry and Nutrition in Metabolic Diseases, Institute for Basic Sciences, Kashan University of Medical Sciences, Kashan, Iran

**Keywords:** Melatonin, Cancer, Angiogenesis, Apoptosis, Autophagy, Endoplasmic reticulum stress, Oxidative stress, Inflammation

## Abstract

Cancers are serious life-threatening diseases which annually are responsible for millions of deaths across the world. Despite many developments in therapeutic approaches for affected individuals, the rate of morbidity and mortality is high. The survival rate and life quality of cancer patients is still low. In addition, the poor prognosis of patients and side effects of the present treatments underscores that finding novel and effective complementary and alternative therapies is a critical issue. Melatonin is a powerful anticancer agent and its efficiency has been widely documented up to now. Melatonin applies its anticancer abilities through affecting various mechanisms including angiogenesis, apoptosis, autophagy, endoplasmic reticulum stress and oxidative stress. Regarding the implication of mentioned cellular processes in cancer pathogenesis, we aimed to further evaluate the anticancer effects of melatonin via these mechanisms.

## Introduction

As the second cause of mortality worldwide, new cases of cancer have recently been reported to increase by 2025 (approximately 19.3 million annually) [1]. Cancer growth control, complete eradication and preventing its incidence are main purposes for cancer-associated investigations. Chemotherapy, radiotherapy and surgery are the major conventional anticancer treatments. The restricted efficiency of these treatments as well as their dangerous side effects have forced researchers to find novel effective anticancer therapies based on herbal extracts and natural compounds as single or combined therapies [2–4].

Melatonin, a multifunctional pleiotropic neurohormone secreted by the pineal gland and other organs including bone marrow, retina, and skin. It is an immune regulatory agent and powerful antioxidant with a capability of preventing cell death in oxidative stress situations. [5, 6]. Moreover, melatonin interrupts cell death mechanisms, inflammation, and redox activity probably resulting in cancer cells sensitization to chemotherapy and radiation [7].

Furthermore, in addition to diverse therapeutic potentials for several diseases [8, 9], melatonin has been shown to possess anticancer abilities against skin cancer [10], glioma [11], lung cancer [12], gastrointestinal cancers [13], gynecological cancers [14, 15], and hematological cancers [16, 17]. Although mechanistic impacts of melatonin on various cancers have been widely demonstrated, in the present review we discuss anticancer effects of melatonin with focusing on molecular pathways including angiogenesis, apoptosis, autophagy, endoplasmic reticulum stress, and oxidative stress.

## Melatonin, a neurohormone with a broad spectrum functions

Monitoring of circadian rhythm is one of the several properties of melatonin, which also possesses oncostatic, vasoregulation, antioxidant, anti-inflammatory, and immunomodulatory abilities [18, 19]. It has been demonstrated that the normally enhanced melatonin levels at night help in the organization of homeostatic metabolic rhythms of targeted organs and systems [20]. Of note, disruption of circadian rhythm has been shown as one of the contributing factors in cancer progression and development [21].

Melatonin, as an antioxidant agent, scavenges free radicals. Melatonin has protective effects on neurodegenerative disorders, epilepsy, and cancer through inhibiting oxidative stress in vitro and in vivo [22, 23]. Melatonin increases the activity and expression of enzymes, including catalase, superoxide dismutase and glutathione peroxidase, implicated in antioxidant abilities [24, 25]. Melatonin also has anti-inflammatory impacts and attenuates pathogenic inflammation through modulating different pathways, including reducing the secretion of tumor necrosis factor-alpha (TNF-α), interleukin-1 (IL-2) and interferon-gamma (IFN-γ), and enhancing the amounts of IL-4, IL-10 and IL-27. Melatonin alleviates pro-inflammatory cytokines secretion via suppressing nuclear factor kappa B (NF-κB) [26–28]. In addition, in neurodegenerative disorders, melatonin blocks cyclooxygenase-2 (COX-2) expression, a pro-inflammatory mediator [29]. Melatonin inhibits apoptosis through regulating Bax/Bcl2 and decreasing caspase-3 activity and expression, proposing that melatonin modulates apoptotic functions in the protection against malignancy and neurodegenerative disorders [30–32].

**Fig. 1:**
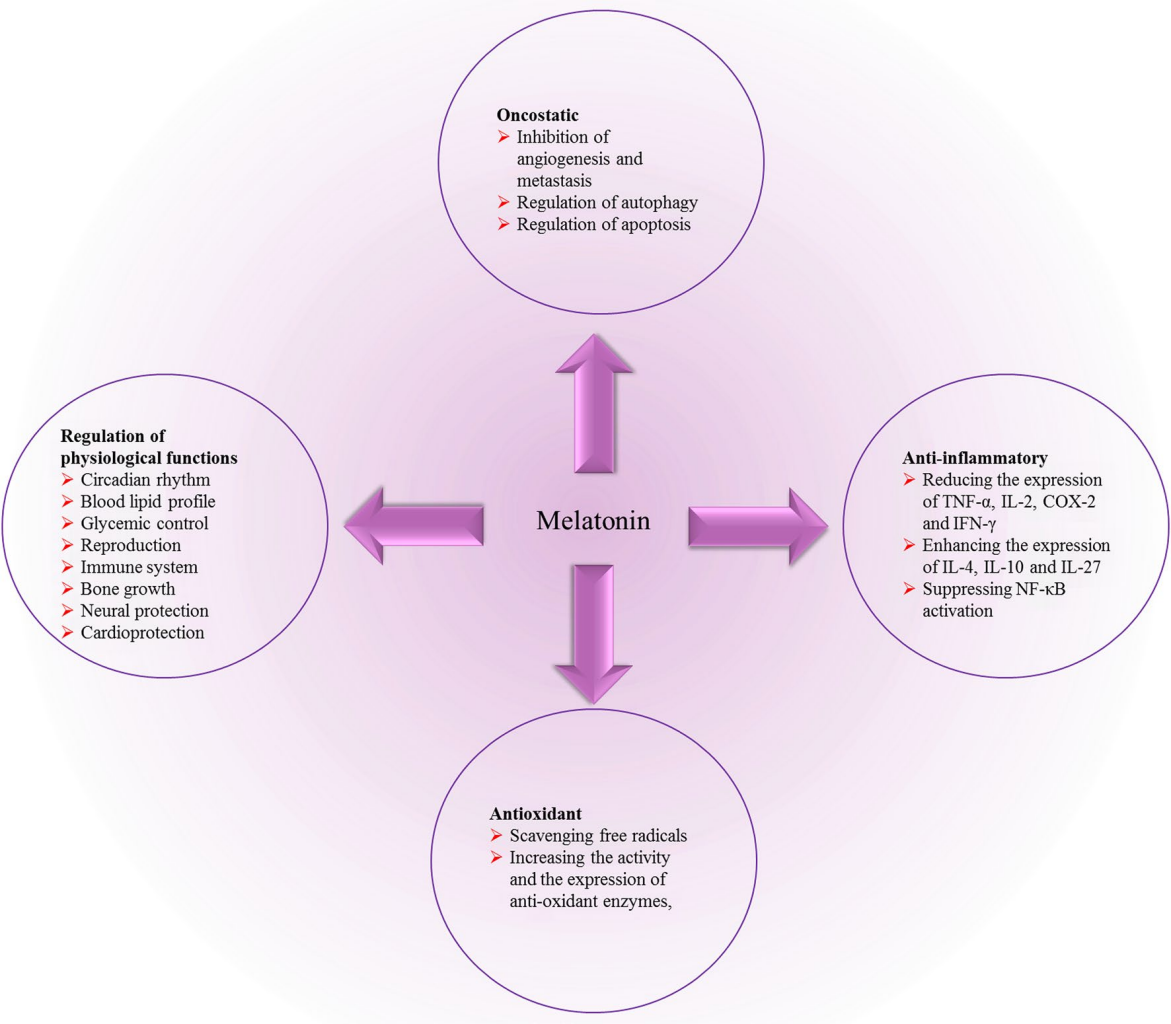
Melatonin with a broad spectrum functions

Melatonin regulates multiple physiological and neural functions (Fig. 1). Among of them, effects on blood lipid profile, glycemic control, gestation, reproduction, and fetal development, neural protection, immune system, and cardiovascular system have been widely documented [33, 34]. Melatonin prevents the growth and promotion of spontaneous and chemically mediated breast tumors [35, 36]. Moreover, at physiological concentrations, melatonin suppresses cell invasiveness and proliferation in breast cancer cells [37].

## Melatonin and cancer: effect on different molecular mechanisms and cellular pathways

**Fig. 2:**
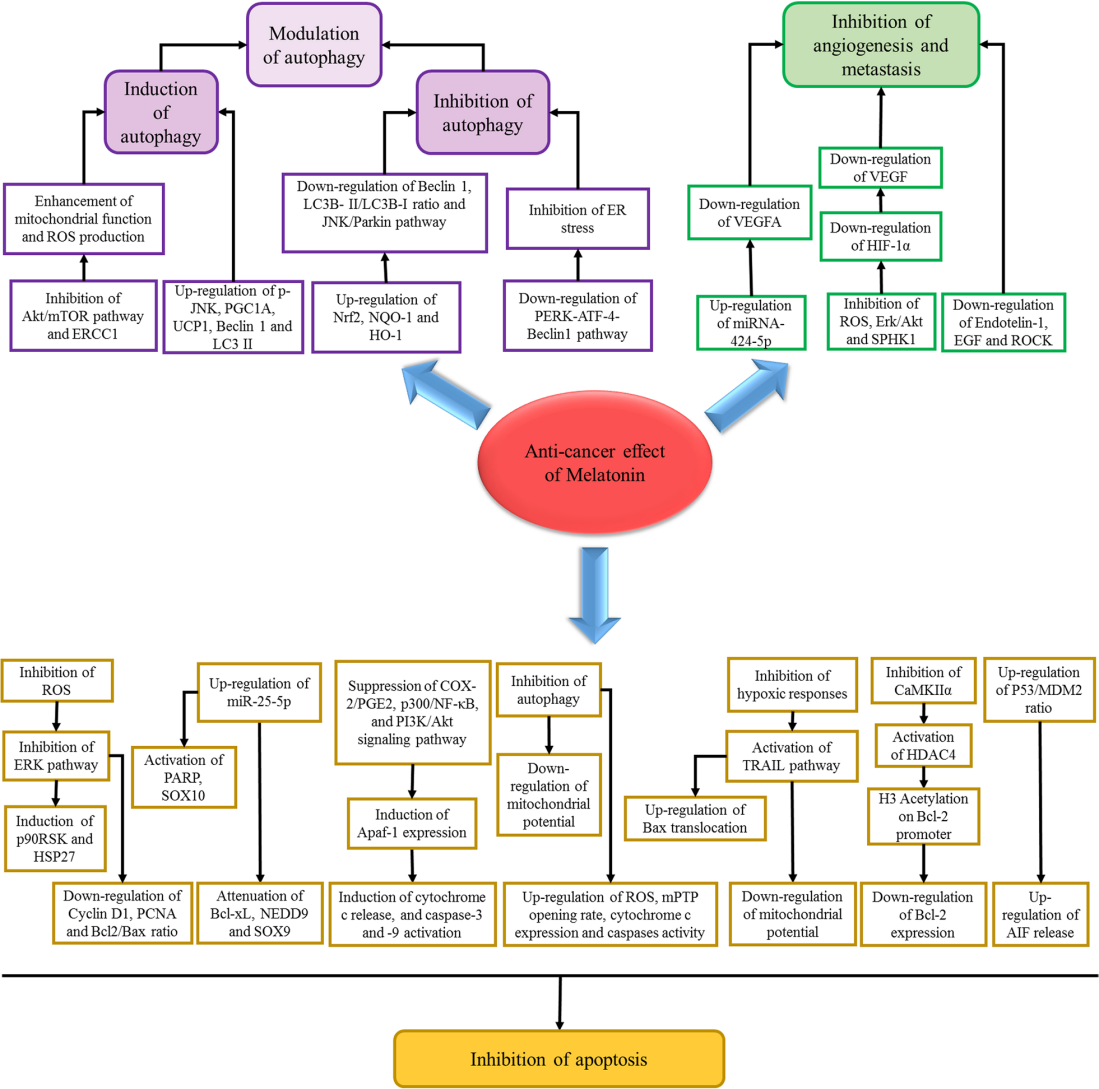
Effects of melatonin on various molecular mechanisms and cellular pathways

In this section we describe the effect of melatonin on oxidative stress and endoplasmic reticulum stress, and various signaling pathways including angiogenesis, apoptosis, autophagy affected by melatonin in different cancer cells (Fig. 2).

### Melatonin and angiogenesis

Angiogenesis is a crucial event implicated in the progression of tumor as well as its metastasis [38]. Hypoxia in the central areas of solid tumor is a leading cause of angiogenesis via activation of angiogenic mediators [38, 39]. Vascular endothelial growth factor (VEGF), the specific mitogen of endothelial cells and the most active pro-angiogenic agent, is a powerful angiogenesis enhancer which increases vascular permeability. Numerous data suggest that, in tumor development, anti-VEGF therapy has important roles in the suppression of tumor cell growth, leading to a considerable amelioration in progression-free survival [40]. Hypoxia-inducible factor-1 (HIF-1) is another key factor in angiogenesis, which modulates hypoxia-activated genes transcription and consists of HIF-1α and HIF-1β heterodimer. The α subunit of HIF-1 is stabilized under hypoxia and degraded under normoxic situations, however, HIF-1β is expressed constitutively [41].

Melatonin has been shown to have regulatory role in angiogenesis process [42]. In other words, melatonin possesses various impacts on neovascularization under diverse pathological and physiological situations. In skin lesions, gastric ulcers, and some physiologic events, melatonin promotes angiogenesis, while in a hypoxic environment, in age-related ocular diseases, and in tumors melatonin suppresses neovascularization in tissues [43].

Melatonin exerts its antitumor potentials via inhibiting HIF-1-induced angiogenesis [44]. Furthermore, melatonin inhibits the accumulation of HIF-1α through suppressing the formation of ROS and the sphingosine kinase 1 (SPHK1) pathway in prostate cancer cells under hypoxic conditions [45]. Melatonin plays an important role in the paracrine interaction between proximal endothelial cells and malignant epithelial cells by a downmodulatory effect on the expression of VEGF in breast tumor cells, which reduces VEGF levels around endothelial cells [46].

Of note, anti-angiogenic potential of melatonin is a key factor resulting in the inhibition of proliferation of cancer cells, as demonstrated in various investigations. For instance, melatonin attenuates proliferation of prostate cancer cells triggered by epidermal growth factor [47]. Melatonin also hampers vasculogenic mimicry of oral cancer cells via inhibition of ROS-activated Akt and ERKs signaling pathway implicating the HIF-α pathway [48]. Melatonin up-regulates TGF-β1 expression in tumor tissues during the inhibition of gastric cancer tumor growth process [49]. Furthermore, apoptotic and anti-proliferative effects of melatonin on breast cancer cells are mediated by the simultaneous activation of the Apaf-1/caspase-dependent apoptotic pathway and the inhibition of PI3K/Akt, p300/NF-κB, and COX-2/PGE2 signaling pathways [32].

Endothelin-1 is a peptide acting as a survival factor in colon cancer, promoting angiogenesis and mediating cell proliferation. Melatonin suppresses endothelin-1 mRNA expression. Also, melatonin blocks the activity of endothelin-1 promoter modulated by NF-κβ and FoxO1 [50]. Melatonin represses ROCK-1, VEGF and HIF-1α genes expressions in oral cancer [51]. Melatonin alters the expression of inflammatory and angiogenic proteins in both co-culture and monoculture of cancer cells and cancer-associated fibroblasts [52]. Melatonin suppresses tumor angiogenesis and the growth of gastric cancer cells in tumor-bearing nude mice. Moreover, melatonin decreases the expression of VEGF and HIF-1α at translational and transcriptional levels within gastric cancer cells during tumorigenesis [53]. Reduced serum levels of VEGF have been reported in cancer subjects treated with melatonin [54]. Vimalraj et al. [55] showed that melatonin upregulates miR-424-5p expression in osteosarcoma cells suppressing VEGFA. Furthermore, it inhibits tumor angiogenesis, regulating surrounding endothelial cells migration and proliferation, and angiogenic growth factors and the morphology of blood vessels E (Table 1).

**Table 1:** Melatonin fights against different cancers trough angiogenesis modulation

Type of malignancy	Melatonin dose/concentration	Angiogenesis-related targets	Key findings	Model	Cell line	Refs.
Breast cancer	1 mM	VEGF, ANG-1, ANG-2	Downregulated angiopoietins with a decrease in VEGF	In vitro	MCF-7	[125]
Dalton's lymphoma	1 and 5 mM	VEGF, FGF, TIMP3	Decreased the Dalton’s lymphoma ascites–mediated angiogenesis	In vivo, in vitro	A549 and SiHa	[126]
serous papillary ovarian cancer	200 µg/100 g b.w	VEGF, HIF-1α	Significantly decreased angiogenesis-associated markers, ovarian cancer size and microvessel density	In vivo	-	[127]
canine mammary tumor cells	1 mM	VEGF	Decreased cell viability, enhanced caspase-3 cleaved and proteins implicated in the apoptotic pathway and diminished pro-angiogenic VEGFA	In vitro	CF-41, CMT-U229	[128]
Neuroblastoma	1 mM or 1 nM	VEGF	Inhibited proliferation and migration of cancer cells	In vitro	SH-SY5Y	[129]
Gastric cancer	3 mM 100, 150 mg/kg	VEGF, HIF-1α, RZR/RORγ, SENP1	Suppressed gastric cancer growth and blockaded tumor angiogenesis Decreased the expression of melatonin nuclear receptor RZR/RORγ	In vitro, in vivo	SGC-7901	[130]
Breast cancer	1 mM	-	Regulated inflammation Decreased cancer cell viability and cancer associated fibroblasts	In vitro	MDA-MB-231	[52]
Breast cancer	1 mM	HIF-1α, VEGF, EGFR, angiogenin,	Reduced protein and gene expression of angiogenesis markers and also decreased cancer cell viability	In vitro	MCF-7, MDA-MB-231	[131]
Breast cancer	10 mg/kg	VEGF	The combination of melatonin and P. acnes cured forty percent of treated mice, suppressed metastasisand decreased angiogenesis and mediated apoptosis	In vivo	EMT6/P	[132]
Prostate cancer	1 mM	HIF-1α, VEGF	Upregulationn of miRNA374b and miRNA3195 mediated melatonin-induced anti-angiogenic properties	In vitro	PC-3	[133]
Oral cancer	1 mM	HIF-1α, VEGF	Decreased cancer cell viability, inhibited metastasis and angiogenesis	In vitro	SCC9, SCC25	[51]
Hepatocellular carcinoma	1 mM	VEGF, HIF-1α, STAT3	Melatonin exerted its anti-angiogenic effects through interfering with the transcriptional activation of mentioned markers	In vitro	HepG2	[134]
Breast cancer	1 mM	VEGF	Inhibited stimulatory impacts on the proliferation of human umbilical vein endothelial cells (HUVECs) as well as VEGF protein levels	In vitro	MCF-7	[46]
Renal cancer	20 mg/kg 10 µM	HIF-1α	Inhibited tumor growth, blocked tumor angiogenesis and diminished HIF-1α protein expression within the tumor mass during tumorigenesis	In vivo, in vivo	RENCA	[135]
Breast cancer	1 mM 40 mg/kg	VEGFR2, micro-vessel density (MVD)	Inhibited tumor growth and proliferation	In vivo, in vitro	MDA-MB-231	[136]
Colon cancer	1 mM	VEGF, HIF-1α	Suppressed invasion and migration	In vitro	HCT116	[44]
Breast cancer	40 mg 0.001 mM, 0.01 mM, 0.1 mM and 1 mM	VEGF, IGF-IR, HIF-1α	Increased miR-152-3p expression leading to suppress breast cancer	In vitro	MDA-MB-468	[137]
Breast cancer	-	IGF-1R, VEGF	Inhibited survival, migration and invasion of breast cancer cells Increased the gene level of miR-148a-3p	In vivo, in vitro	MDA-MB-231	[138]
Advanced cancer patients (CRC, HCC, RCC, NSCLC)	20 mg	VEGF	Controlled tumor growth by anti-angiogenic roles	Human	-	[54]

### Melatonin and oxidative stress

In normal cellular condition, there is a balance between the production of oxidants, so called reactive oxygen species (ROS), and their neutralizing compounds, named antioxidants. The state of excess ROS, in which the oxidant content of the cells dominates the neutralizing capacity of antioxidants, is defined as oxidative stress [56, 57]. Sustained oxidative stress increases the risk of cancer development either through inducing mutagenesis or by promoting the expression of proto-oncogenes such as cyclin D1. It also plays a signaling role in the activation of several genes involved in the cancer progression including the mitogen-activated protein kinase (MAPK), extracellular signal-regulated kinase (ERK) and JUN N-terminal kinase (JNK) [58, 59].

Melatonin role as a natural ally against oxidative stress has been revealed in many in vitro and in vivo studies. Detoxification of oxidants by melatonin is triggered by several direct or indirect mechanisms. In direct scenario, melatonin neutralizes the oxidants by its nonreceptor‐mediated free radical scavenging capacity. As indirect scenario, melatonin reduces the oxidative content through several mechanisms such as activating anti-oxidative enzymes and suppressing pro‐oxidative enzymes. It also stabilizes the mitochondrial inner membrane, thereby maintaining mitochondrial integrity leading to a reduced electron leakage and ROS generation [60, 61].

The inducing role of oxidative stress in cancer progression and preventive role of melatonin in the production and function of oxidants indicated a possible oncostatic property for melatonin [62]. Subsequently, it was revealed that melatonin reduces the oxidative damage to cellular components under conditions where toxic oxygen derivatives are acknowledged to be released [63, 64]. Moreover, in vitro studies demonstrated that melatonin treatment reduces the amount of oxidative contents in a variety of cancer cells, which was further supported by in vivo studies (Table 2).

**Table 2:** Melatonin acts as an antioxidant in cancer models

Cancer	Melatonin dose/concentration	Key findings	Model	Cell line/animal	Refs.
Breast cancer	1 μM 5, 10, 50 mg/kg	Limited paclitaxel-mediated mitochondrial dysfunction and protected against paclitaxel-mediated neuropathic pain	In vitro, in vivo	MCF-7 Male and female Sprague Dawley rats	[198]
Neuroblastoma	10 μM	Reduced oxaliplatin-induced neurotoxicity	In vitro	SH-SY5Y	[199]
Breast cancer	0.3 mM	Supported doxorubicin effects by apoptosis and TRPV1activation, and through mediating cancer cell death	In vitro	MCF-7	[200]
Cervical cancer	1 mM	Enhanced cisplatin-mediated cytotoxicity and apoptosis	In vitro	HeLa	[163]
Lung cancer	1 nm, 1 μm, 1 mm	Exerted immunomodulatory effects	In vitro	SK‐LU‐1	[201]
Pancreatic cancer	26.8 mg	capecitabine and melatonin provided an amelioration in antioxidant status and synergistic antitumoral effects	In vivo	Male Syrian hamsters	[202]
Leukemia	1 mM	Protected healthy cells from chemotherapy-mediated ROS production and induced tumor cell death	In vitro	HL-60	[180]
Hepatocellular carcinoma	1, 100 μM	The responses of angiogenic chemokine genes to melatonin were determined by the characteristics of cancer cells	In vitro	HCC24/KMUH,	[203]
Pancreatic cancer	53.76 mg	Exerted more potent beneficial effects than celecoxib on the decrease in tumor nodules, oxidative stress and death	In vivo	Male Syrian hamsters	[204]
Breast cancer	2.5 mg/kg	Antioxidant effects	In vivo	Female Sprague Dawley rats	[205]
Pancreatic cancer	26.88, 53.76 mg	Decreased oxidative damage and cancer nodules mediated by BOP in the pancreas	In vivo	Male Syrian hamsters	[206]
Cervical cancer	10–1000 μM	This study showed melatonin effects on radiotherapy is dose-dependent	In vitro	HeLa	[207]
Hepatocellular carcinoma	20 mg/kg	Fostered the survival and therapeutic potential of MSCs in HCC, by inhibition of oxidative stress and inflammation as well as apoptosis induction	In vivo	Adult female rats	[120]
Cervical cancer	10 μM	Enhanced TNF-α-mediated cervical cancer cells mitochondrial apoptosis	In vitro	HeLa	[14]
Bladder cancer	1 mM 100 mg/kg	Inhibited the growth, migration, and invasion of cancer cells	In vivo, in vitro	HT1197, HT1376, T24, RT4 Male C57B/L6 mice	[208]
Lung cancer	0.25–2.5 mM	Enhanced palladium-nanoparticle-induced cytotoxicity and apoptosis	In vitro	A549, H1299	[209]
Lymphoma, cervical cancer, hepatoblastoma, gastric cancer, breast, colon and lung adenocarcinoma,	0–2 mM	Sensitizees shikonin-mediated cancer cell death induced by oxidative stress	In vitro	U937, HeLa, Hep-G2, AGS, MCF-7, SW480, A549	[210]

### Melatonin and endoplasmic reticulum stress

Endoplasmic reticulum (ER) is an entry site for secretory proteins and most integral membrane proteins for proper folding and covalent modifications to assemble into a functional complex. In addition to the processing of proteins, ER is involved in various cellular functions including lipid synthesis, fatty acid turnover, detoxification, Ca^2+^ homeostasis. The ER network extends into all cell compartments to sense intrinsic and extrinsic perturbations and integrate the stress signals for maintenance of cellular homeostasis to preserve proper cellular and organismal function [65, 66]. However, the ER function can be impacted and disturbed by a multitude of exogenous and endogenous factors, leading to the accumulation of mis/unfolded proteins in the ER. This causes the imbalance between the client proteins load in the lumen of ER and the folding capacity of this organelle leading to the failure of the ER to cope with unusual high protein folding load, which is termed 'ER stress' [67]. To restore protein homeostasis, an adaptive signal transduction pathway called the unfolded protein response (UPR) is activated to induce compensatory responses to stressors for recovering normal ER function [68]. Signaling proteins sensing UPR include inositol-requiring protein-1α (IRE1α), activating transcription factor 6α (ATF6α) and protein kinase RNA (PKR)-like ER kinase (PERK). In nonstressed cells, UPR stress sensors are maintained in an inactive state through direct binding to the ER chaperone proteins, Bip (78-kDa glucose regulated protein, GRP78). Upon ER stress, aggregation of misfolded proteins leads to the dissociation of UPR sensors from Bip, which causes activation of UPR signals [69]. Although activation of UPR signaling pathways is a cellular strategy to increase survival, this pathway will instead activate cell death signaling pathways when the intensity or duration of cellular stress increases. Therefore, certain anti-cancer patterns may activate ER stress/UPR pathway to induce apoptosis in cancer cells [70].

Melatonin induces mitochondria-mediated apoptosis in colorectal cancer cells through reducing the expression of PrP^C^ and PINK1 resulting in the enhancement of superoxide production and induction of ER stress [71]. Melatonin also ameliorates ER-stress mediated insulin resistance. ER stress induces autophagy in pancreatic β cells, which this pathway plays an important role in insulin production and secretion. In the glucose analog 2-DG-treated rat insulinoma INS-1E cells, melatonin reduces insulin production via ER stress-induced autophagy [72]. Combination of melatonin with ER stress inducer tunicamycin increases the sensitivity of cancer cells to apoptosis through inhibiting the expression of COX-2 and increasing the Bax/Bcl-2 ratio and CHOP levels [73]. Selective inhibition of ATF-6 by melatonin results in the suppression of COX-2 production and enhancement of cancer cells to ER-stress induced apoptosis [74].

Melatonin increases apoptosis through enhancing caspase-3, -8 and -9 activities, Bax/Bcl-2 ratio, PARP cleavage and cytochrome c, p53 and Fas-L proteins concentrations in hepatocellular carcinoma, which this effect is mediated by the elevation of ER stress characterized by up-regulation of ATF6, CHOP and Bip [75]. Furthermore, melatonin increases the sensitivity of hepatocellular carcinoma cells to sorafenib through targeting the PERK-ATF4-Beclin1 pathway [76]. The same results have been reported in gastric cancer; melatonin inhibits cell proliferation through inducing activation of the IRE/JNK/Beclin1 signaling [77].

Melatonin in combination with the ER stressor thapsigargin increases the expression level of nuclear mammalian RNA-binding protein (HuD) resulting in the reduction of intracellular biosynthesis of insulin. Suppression of AKT/PI3K pathway and induction of nuclear mTOR (Ser2481, Ser2448) expressions by melatonin sensitizes rat insulinoma INS-1E cells to insulin through increasing the expression of insulin receptor substrate [78]. In contrast with these reports, melatonin has been reported to inhibit tunicamycin-induced ER stress in human hepatocellular carcinoma cells and increase the response of these cells to cytotoxic effects of doxorubicin; this is accompanied by inhibition of the PI3K/AKT pathway, elevation of CHOP and reduction of Survivin [79]. These evidences suggest that melatonin could improve the toxic effect of anti-cancer agents on cancer cells through regulating ER stress in cells (Table 3).

**Table 3:** Melatonin suppressive effects on various cancers via regulation of ER stress

Cancer	Melatonin dose/concentration	Effect on ER stress	Key findings	Model	Cell line/animal	Refs.
Gastric cancer	1, 2, 3 mM 50 mg/kg	Activate	Melatonin-mediated inhibition of cancer cell proliferation is induced by the IRE/JNK/Beclin1 signaling activation	In vitro, in vivo	AGS, SGC-7901 cells Male BALB/c nude mice	[77]
Lung, liver and cervical cancer	2 mM	Activate	Induced apoptosis by ROS generation and JNK activation	In vitro	HepG2, A549, HeLa	[211]
Hepatocellular carcinoma	10^–5^ M	-	enhanced HCC sensitivity to sorafenib through suppressing autophagy	In vitro	HepG2, 7721, Huh7, LO2	[76]
Colorectal cancer	0–1 mM	Activate	Induced mitochondria-induced cellular apoptosis	In vitro	SNUC5/ WT	[71]
Hepatoma	10^–7^-10^–3^ mM	-	Melatonin was shown as a novel selective ATF-6 inhibitor that can sensitize human hepatoma cells to ER stress inducing apoptosis	In vitro	HepG2	[74]
Insulinoma	100 μM	Activate	Melatonin-induced insulin synthesis involved autophagy and EDC3 protein in rat insulinoma cells and subsequently resulted in a resuction in intracellular production of insulin	In vitro	INS-1E	[72]
Hepatocellular carcinoma	1 mg/kg/d	Activate	Activated ER stress and apoptosis	In vivo	Male Wistar rats	[75]
Insulinoma	10, 50 μM	-	Decreased nuclear and cellular expressions of p85α Decreased cellular expression of HuD and led to a reduction in cellular insulin level and rise in insulin secretion	In vitro	INS-1E	[78]
Hepatocellular carcinoma	10^–3^ M	Inhibit	Attenuated ER stress-mediated resistance to doxorubicin by downregulating the PI3K/AKT pathway, enhancing CHOP levels and reducing Survivin levels	In vitro	HepG2, SMMC-7721	[79]
Hepatoma	10^–9^, 10^–7^, 10^–5^, 10^–3^ μM	Activate	Sensitized cancer cells to ER stress-mediated apoptosis by downregulating COX-2 expression, enhancing the levels of CHOP and reducing the Bcl-2/Bax ratio	In vitro	HepG2, HL-7702	[73]

### Melatonin and autophagy

Autophagy is a complicated process maintaining intracellular homeostasis by eliminating degraded proteins and organelles during cellular stress. Autophagy is principally considered as a pro-survival process, but, excessive or inappropriate autophagy contributes to the cell death, a process known as autophagic cell death or type II programmed cell death [80].

Autophagy is a complicated process, which consists of five sequential steps, including: (a) initiation complex formation and double-membrane phagophore (nucleation) maturation; (b) membrane elongation and autophagosome formation sequestering cargo; (c) fusion with lysosome; (d) inner membrane disruption leading to degradation of cargo by hydrolases; and (e) macromolecular component utilization [81]. These steps of the autophagy pathway are regulated by more than 35 autophagy related genes (ATGs) and proteins most of which function in complexes. The initiation phase is regulated by Unc-51-like kinase1 (mammalian homologues of Atg1, ULK1)–Atg13–Atg101–FIP200 (mammalian homologues of Atg17) protein complex. Unc-51-like kinase1 phosphorylates and activates Beclin-1 (mammalian homologue of Atg6). Beclin-1 is a part of multiprotein-complex, class III PI3-kinase Vps34–p150 (mammalian homolog of Vps15)–Atg14-like protein (Atg14L)–Beclin-1, promoting nucleation [81–83]. The elongation phase is regulated by two ubiquitin-like conjugation systems, Atg12 and LC3 (mammalian homologue of Atg8). In the first conjugation system, Atg12 is activated by Atg7 (E1-like enzyme), transferred to Atg10 (E2-like enzyme) and conjugated to Atg5. The Atg12–Atg5 conjugates further couples with Atg16 (*Atg16L* in mammals) to form the E3-like complex. In the LC3 conjugation system, LC3 is cleaved by a cysteine protease, Atg4, forming LC3-I. Thereafter, LC3-I is activated by Atg7 (E1-like enzyme), transferred to Atg3 (E1-like enzyme) and conjugated to phosphatidylethanolamine (PE) to form LC3-II; this process is facilitated by the E3-like complex. This lipidated form of LC3, LC3-II, is recruited to the autophagosome membrane. Finally, the Atg9 dependent pathway promotes autophagosome membrane expansion [81–83].

Cargo sequestration can be selective or non-selective; the selectivity is based on autophagy receptors such as P62/SQSTM1, NBR1, NDP52, NIX/BNIP3L, BNIP3 and FUNDC1 [82]. Fusion of autophagosome with lysosome is the next step. The inner vesicle is degraded by lysosomal hydrolases, including cathepsin B, D (a homolog of proteinase A), and L. The degradation products are released to the cytosol and used in different anabolic pathways [84]. ER stress-induced activation of UPR pathways promotes induction of autophagy [85]. Activated PERK/ATF4 pathway up-regulates the expression of ATG genes including ATG5, ATG7, and ATG10 [86]. The conversion of LC3-I conversion to LC3-II is also induced by PERK pathway [87]. Activation of IRE1α pathway induces the expression of Beclin1 and the phosphorylation of Bcl-2 by JNK, which subsequently results in the Bcl-2–Beclin 1 dissociation [88–90]. The release of Ca^2+^ from ER to cytosol triggers autophagy pathway through activating several mechanisms including (I) inhibition of mTOR by Ca^2+^/calmodulin dependent kinase kinase-β-mediated activation of AMP-activated protein kinase (AMPK) [91], and (II) dissociation of Bcl-2–Beclin 1 by inducing death-associated protein kinase (DAPK) 3-mediated Beclin 1 phosphorylation [92].

Melatonin has a modulatory effect on autophagy in various cell types and different conditions. Melatonin indirectly modulates autophagy through affecting oxidative stress, ER stress and inflammation [69]. Melatonin enhances the effectiveness of cisplatin and radiotherapy in head and neck squamous cell carcinoma, which this effect is mediated by the excessive activation of mitochondria leading to the over-production of ROS and subsequent induction of autophagy and apoptosis [93]. Melatonin also increases cytotoxic effects of rapamycin in cancer cells. Combination of rapamycin and melatonin suppresses AKT/mTOR pathway activation, which this effect leads to the enhancement of mitochondrial function and ROS production resulting in the induction of apoptosis and mitophagy [94]. Melatonin induces autophagy in clear cell renal cell carcinoma through activating transcriptional coactivator peroxisome proliferator-activated receptor gamma coactivator 1A (PGC1A) and uncoupling protein 1 (UCP1); this is associated with the elimination of lipid deposits without generating ATP, which subsequently leads to the tumor size reduction [95].

Melatonin reduces the viability liver cancer cells through transient induction autophagy by up-regulating JNK phosphorylation. However, ATG5 silencing sensitizes cancer cells to melatonin-induced apoptosis. This suggests that modulation of autophagy by melatonin has dual effect on cell death [96]. Similarly, disruption of autophagy sensitizes glioblastoma cells and tongue squamous cell carcinoma to melatonin-induced apoptosis [97]. Melatonin-induced autophagy is suggested to be mediated by activation of melatonin membrane receptor in tongue squamous cell carcinoma and suppression of melatonin membrane receptor-dependent autophagy may be strategy for treatment of tongue squamous cell carcinoma [98].

Several studies indicate that melatonin may induce apoptosis in cancer cells through inhibiting autophagy pathway. Melatonin down-regulates Beclin-1 and p62 expressions and LC3B-II/LC3B-I ratio in colitis-associated colon carcinogenesis in mice; this effect is associated with the increased level of Nrf2 and its downstream antioxidant enzymes including NAD(P)H:quinone oxidoreductase (NQO-1) and heme oxygenase-1 (HO-1). These suggest that the ameliorative effect of melatonin on inflammation and oxidative stress results in the reduction of autophagy [99]. Induction of ER stress is associated with the activation of autophagy in sorafenib-treated hepatocellular carcinoma cells, which this contributes to the resistance of cancer cells to apoptosis. Combination of melatonin with sorafenib inhibits ER stress-related autophagy through suppressing the PERK-ATF4-Beclin1 pathway leading to the sensitivity of hepatocellular carcinoma cells to sorafenib [76]. Co-stimulation of cancer cells with cisplatin and melatonin induce apoptosis in HeLa cells, which this effect is accompanied by inactivating mitophagy via blockade of JNK/Parkin pathway [100]. In contrast with this report, melatonin has been found to reversed the effects of cisplatin in HepG2 cells through suppression of mTOR and DNA excision repair cross complementary 1 (ERCC1) proteins expressions and up-regulation of Beclin-1 and LC3II expressions [101]. Taken together, different effects of melatonin on autophagy may be related to type of cancer cells, the stage of cancer and dose of melatonin (Table 4).

**Table 4:** The effect of melatonin on autophagy machinery in recently reported findings

Cancer	Melatonin dose/concentration	Autophagy-related targets	Effect on autophagy	Key findings	Model	Cell line	Refs.
Lung, liver and cervical cancer	2 mM	LC3	Activate	Induced apoptosis by ROS generation and JNK activation	In vitro	HepG2, A549, HeLa	[124]
Glioblastoma	1 mM	Beclin 1, LC3-II	Activate	Autophagy disruption stimulated the melatonin-mediated apoptosis in cancer cells	In vitro	A172, U87-MG	[97]
Uterine leiomyoma	25 mg/kg 0.1, 0.5, 1, 1.5, 2 mM	Beclin1 and LC3	Activate	Reduced tumor growth and proliferation	In vivo, in vitro	ELT3 cells, orthotopic uterine leiomyoma mouse model	[192]
Hepatocellular carcinoma	10^−5^_ 10^–3^ M	PERK-ATF4-Beclin1 pathway	Inhibit	enhanced HCC sensitivity to sorafenib through suppressing autophagy	Human	-	[76]
Colorectal cancer	10 μM	LC3-II	Activate	Induced interplay of apoptosis, autophagy, and senescence	In vitro	HCT116	[171]
Clear cell renal cell carcinoma	200 mg/kg 0.5, 1, 2 μM	PGC1A, UCP1, LC3‐II	Activate	Melatonin/PGC1A/UCP1 promoted tumor slimming and repressed tumor progression through initiating autophagy and lipid browning	In vivo, in vitro	HK2, 786‐O, A498, Caki‐1, and ACHN cells Mice	[95]
Neuroblastoma	0.1‐ 10 nM 40‐80 mg/kg	LC3II	Activate	Promoted cancer cell differentiation through activation of hyaluronan synthase 3-mediated mitophagy	In vivo, in vitro	N2a N2a‐allografted nude mice	[193]
Head and neck squamous cell carcinoma	0.1, 0.5, 1, 1.5 mM	ATG12-ATG5	Activate	Induced intracellular ROS	In vitro	Cal-27, SCC-9	[93]
Hepatocellular carcinoma	1 mM	mTOR, Beclin-1	Activate	Decreased cisplatin-mediated cell death by a counter-balance between the roles of apoptotic- and autophagy-related proteins	In vitro	HepG2	[101]
Hepatocellular carcinoma	2 mM	Beclin-1, p62, LC3II, LAMP-2	Activate	Ceramide metabolism regulated apoptotic and autophagy cell death mediated by melatonin	In vitro	HepG2	[96]
Neuroblastoma	1 μM	Beclin‐1, LC3‐II	Activate	Enhanced autophagic activity by the SIRT1 signaling	In vitro	SH‐SY5Y	[194]
Gastric cancer	10^−4^ M	LC3	Activate	Hyperbaric oxygen sensitized cancer cells to melatonin-mediated apoptosis	In vitro	SGC7901	[151]
Colon cancer	1 mg/kg	Beclin-1, LC3B-II/LC3B-I ratio, p62	Inhibit	Decreased autophagy by improving oxidative stress and inflammation	In vivo	Male Swiss Albino mice	[99]
Glioblastoma	1 mM	LC3, Beclin-1	Activate	Inhibited tumor bulk proliferation, and enhanced chemotherapy effects	In vitro	Glioblastoma-initiating cells	[195]
Oral cancer	0.5–2 mM	LC3-II	Activate	Decreased drug resistance, and induced autophagy and apoptosis	In vitro	SAS, SCC9, SASV16, SASV32, SCC9V16, SCC9V32	[139]
Gastric cancer	50 mg/kg 1, 2, 3 mM	p62, Beclin-1, LC3A/B-II	Activate	Melatonin-mediated inhibition of cancer cell proliferation is induced by the IRE/JNK/Beclin1 signaling activation	In vivo, in vitro	AGS, SGC-7901 Male BALB/c nude mice	[77]
Hepatocellular carcinoma	10, 20 mg/kg 100 μM	Beclin-1, LC3-I/LC3-II	Activate	Induced protective autophagy preventing hepatoma cells from undergoing apoptosis	In vitro, in vivo	H22	[196]
Insulinoma	100 μM	LC3II	Activate	Melatonin-induced insulin synthesis involved autophagy and EDC3 protein in rat insulinoma cells and subsequently resulted in a resuction in intracellular production of insulin	In vitro	INS-1E	[72]
Chriocarcinoma	1 mM	LC3B	Inhibit	Modulated autophagy and the Nrf2 pathway in normal vs. tumor trophoblast cells, being cytoprotective in normal cells whilst enhancing apoptosis in tumoral trophoblast cells	In vitro	BeWo	[197]
Cervical cancer	1 mM	JNK/Parkin	Inhibit	Sensitized cancer cells to cisplatin-mediated apoptosis by suppression of JNK/Parkin/mitophagy pathways	In vitro	HeLa	[100]
Head and neck squamous cell carcinoma	0.1, 0.5 or 1 mM 300 mg/kg	LC3-II, Nix	Activate	Enhanced ROS production, increased apoptosis and mitophagy, and could be used as an adjuvant agent with rapamycin	In vitro, in vivo	Cal-27, SCC-9 Harlan Sprague–Dawley mice	[94]
Tongue squamous cell carcinoma	0, 0.5, 1, 2 mM 100 mg/kg	LC3, ATG7	Activate	Suppression of MT2-TFE3-dependent autophagy enhanced melatonin-mediated apoptosis	In vitro, in vivo	Cal27, SCC9 Male athymic nude mice	[98]

### Melatonin and apoptosis

The balance between cell proliferation and death in tissues is maintained by apoptosis, a classical form of programmed cell death. Apoptosis is associated with the disassembly of apoptotic cells into membrane-enclosed vesicles, which are removed by macrophages without inducing inflammatory responses. Apoptosis is mediated by two principle signaling pathways, including extrinsic and intrinsic pathways [102]. The extrinsic apoptotic signaling pathways, defined as death receptor pathways, are initiated by the interaction of transmembrane death receptors (Fas, TNFR1, DR4 and DR5) with extracellular ligands (FasL, TNFα, TRAIL, and TNFSF10) resulting in the activation of adaptor proteins such as Fas-associated death domain (FADD). Activated FADD recruits initiator caspases (caspase 8 and caspase 10) to form the death-inducing signal complex (DISC). Formation of DISC leads to the proteolytic activation of caspase 8, which is the main initiator caspase of the extrinsic apoptotic signaling pathway. Caspase 8 activates executioner caspases (caspase 3, caspase 6, and caspase 7) and cleaves Bid, a BH3-only domain member of the B cell lymphoma-2 (Bcl-2) family. Truncated Bid (tBid) translocates to mitochondria and activates other proapoptotic Bcl-2 family members including Bak or Bax [102, 103].

The intrinsic apoptosis pathway, defined as mitochondrial-mediated apoptotic pathway, is activated by exogenous and endogenous stimuli such as DNA damage, oxidative stress, chemotherapy and radiotherapy. This apoptosis pathway is mediated by insertion of pro-apoptotic Bcl-2 family members (Bax/Bak) into mitochondrial membrane leading to the mitochondrial outer membrane permeabilization and release of pro-apoptotic factors such as cytochrome c, Smac/DIABLO, the nuclease EndoG, the oxidoreductase AIF, and the protease HtrA2/Omi [104]. Therefore, activation of pro-apoptotic Bcl-2 family members (Bax/Bak) is essential for cancer therapy. In contrast, elevation of anti-apoptotic Bcl-2 family proteins inhibits apoptosis in cancer cells through heterodimerization with Bax/Bak preventing the release of pro-apoptotic factors from mitochondria; this could result in the resistance of cancer cells to immune-surveillance [105, 106]. Once in the cytosol, cytochrome c combines with Apaf–1 and procaspase-9 to drive the assembly of the apoptosome; this molecular platform activates caspase 9, which this is followed by the activation of caspase-3 cascade of apoptosis [107]. Smac/DIABLO and HtrA2/Omi induce apoptosis through degrading inhibitor of apoptosis protein (IAP) family, neutralizing the inhibitory effect of IAPs on caspases [108]. The nuclease EndoG and the oxidoreductase AIF translocate to the nucleus, where they trigger internucleosomal DNA fragmentation independently of caspases [109].

As mentioned earlier, UPR signaling may promote the apoptotic pathways. Upon ER stress, apoptosis signal-regulating kinase 1 (ASK1) is recruited by IRE1α-TNF receptor-associated factor 2 (TRAF2) complex, causing activation of ASK1 and the downstream JNK pathway. Activation of JNK results in the phosphorylation of Bcl-2 and Bax; phosphorylation of Bcl-2 family suppresses antiapoptotic activity of Bcl-2, while induces mitochondrial translocation of Bax and activation of apoptosis pathway. Activated JNK also activates C/EBP homologous protein (CHOP), a stress-induced transcription factor inducing the expression of pro-apoptotic Bcl-2 family members. Furthermore, IRE1α-TRAF2 complex triggers the activation of caspase-12, which this caspase translocates from the ER to the cytosol, where it activates caspase-9, independent from the apoptosome pathway [110]. Furthermore, Activated PERK phosphorylates eIF2α, promoting the expression of activating transcriptional factor 4 (ATF4); ATF4 translocates to the nucleus where it induces CHOP expression [111].

Melatonin is reported to restrict tumor growth and cancer cell proliferation through inducing apoptosis in cancer cells (Table 5). As a powerful antioxidant melatonin inhibits ROS-induced activation of extracellular-regulated protein kinases (ERKs) and Akt pathways which are involved in the cancer cell survivor; inactivation of ROS-dependent Akt signaling contributes to the down-regulation of cyclin D1, PCNA, and Bcl-2 and up-regulation of Bax in cancer cells [48]. Inhibition of MDM2 expression is a mechanism by which melatonin induces apoptosis through upregulating the activity of caspase-3 and -9; MDM2 is an E3 ubiquitin ligase, which negatively regulates the p53 tumor suppressor [112, 113]. Under hypoxic conditions, tumor cells become resistant to TRAIL-induced cell apoptosis; this contributes to the up-regulation of anti-apoptotic protein expression and reduction of pro-apoptotic protein expression. Treatment with melatonin blocks hypoxic responses leading to the induction of apoptosis in TRAIL resistance tumor cells by the regulation of mitochondrial transmembrane potential and induction of Bax translocation [114]. Melatonin inhibits cancer cell growth by increasing cell cycle arrest in the G2/M phase, which this effect is coincident with the induction of apoptosis through up-regulating the expression of p53, p21, caspase-3/8/9, PARP, cytochrome c, Bax, JNK 1,-2 and -3 and p38 MAPKs in cancer cells [115]. Melatonin triggers two distinct apoptotic processes including TGFβ1 and caspase-independent early apoptosis and TGFβ1 and caspases-dependent late apoptosis. Early apoptosis is associated with the elevation level of p53/MDM2 ratio and up-regulation of AIF release; this process is independent to caspase activity or cleavage of PARP. Late apoptosis is associated with elevation of caspases-9 and -7 activity and cleaved-PARP level as well as reduction of Bcl-2/Bax ratio [116]. Melatonin also induces apoptosis through simultaneous suppression of COX-2/PGE2, p300/NF-κB, and PI3K/Akt signaling pathway. Inhibition of these pathways leads to the induction of Apaf-1 expression triggering cytochrome *c* release, and caspase-3 and -9 activation and cleavage [32].

**Table 5:** Anticancer effects of melatonin by apoptosis induction in experimental investigations

Cancer	Melatonin dose/concentration	Apoptosis-related targets	Key findings	Model	Cell line	Refs.
Oral cancer	0.5–2 mM	caspase-3, caspase-9, PARP	Decreased drug resistance, and induced autophagy and apoptosis	In vitro	SCC9V32, SCC9V16, SASV32, SASV16, SAS, SCC9	[139]
Lung cancer Hepatocellular carcinoma Cervical cancer	2 mM	caspase-3, PARP, Bax, Bcl-2	Decreased cell viability and increased LDH release	In vitro	Hela A549 HepG2	[124]
Glioblastoma	1 mM	Bax, Bcl-2	Induced apoptosis and autophagy	In vitro	A172 U87-MG	[97]
Colorectal cancer	0.5, 1 mM	caspase-3, PARP, NEDD9, SOX9, Bcl-xL, SOX10	Enhanced apoptosis through miR-25-5p induced NEDD9 suppression in cancer cells	In vitro	CCD-18Co, HT29, SW480, HCT116	[121]
Breast cancer	3.5–20 mM 2 mg/kg	caspase-3	Repressed drug resistance through apoptosis induction and angiogenesis inhibition	In vitro, in vivo	EMT6/CPR, EMT6/VCR/R	[140]
Lung cancer	2, 4, 6 mM	HDAC9	HDAC9 knockdown increased the anticancer potentials of melatonin	In vitro, in vivo	A549, H838, H1299, and Calu-1	[118]
Ehrlich carcinoma	20 mg/kg	Bcl‐2, caspase-3, caspase-9,	Inhibited the proliferation and growth of tumor via inducing apoptosis and through suppressing tumor vascularization	In vivo	EAC	[141]
Head and neck squamous cell carcinoma	0.1, 0.5, 1, and 1.5 mM	Bax, Bcl-2	Potentiated the cytotoxic impacts of radiotherapy and CDDP, and induced intracellular ROS leading to mitochondria-induced autophagy and apoptosis	In vitro	SCC-9, Cal-27	[93]
Hepatocellular carcinoma	20 mg/kg	Caspase-3, Bax, Bcl-2, survivin	Fostered the survival and therapeutic potential of MSCs in HCC, by inhibition of oxidative stress and inflammation as well as apoptosis induction	In vivo	-	[120]
Cervical cancer	10 μM	CaMKII/Parkin/mitophagy, caspase-3, caspase-9	Enhanced TNF-α-mediated cervical cancer cells mitochondrial apoptosis	In vitro	HeLa	[119]
Gastric cancer	3 mmol/L	Caspase 9, Caspase 3, AKT, MDM2	Promoted apoptosis through downregulation of MDM2and AKT	In vitro	AGS, MGC803	[112]
Melanoma	1 M 25 mg/kg	cytochrome c, caspase-3, caspase-9, Bcl-2	Synergized the antitumor effects of vemurafenib through suppressing cell proliferation and cancer-stem cell traits by targeting NF-κB/iNOS/hTERT signaling	In vitro, in vitro	G361, A431, A375, SK-Mel-28	[142]
Breast cancer	1 mM	caspase-3	Increased apoptosiss and decreased proliferation in cancer cells	In vitro	MDA-MB-231, MCF-7	[143]
Pancreatic cancer	10^–10^, 10^–12^ M	Bax, Bcl-2, caspase-3, caspase-9	Improved the anti-tumor effects of gemcitabine through apoptosis regulation	In vitro	PANC-1	[144]
Breast cancer	25 µM	Bax, Bcl-2	Decreased the cell proliferation and increased apoptosis and differentiation in cancer cells	In vitro	MCF-7, HEK293	[145]
Leukemia	1 mM	Bcl-2, Bcl-xL	Synergistic effect on chemotherapeutic agent	In vitro	HL-60	[146]
Breast cancer	0.1–5 mm 1 mg/kg	-	Melatonin caused apoptosis induction, angiogenesis inhibition, and activation of T helper 1	In vitro, in vivo	EMT6/P	[147]
Colorectal cancer	1 mM	BAX, caspase3, PARP1	Induced mitochondria-induced cellular apoptosis	In vitro	SNUC5/WT	[71]
Breast cancer	1 nM and 100 nM	c-IAP1, XIAP, survivin, MCL-1, BCL-2,	Enhanced cytotoxic effects of arsenic trioxide and apoptosis induction	In vitro	MCF-7	[148]
Pancreatic cancer	0.1, 1, or 2 mM 40 mg/kg	*cytochrome c* XIAP, Mcl-1, Survivin, Bcl-2, PARP	Reinforced the anticancer effect of sorafenib via downregulation of PDGFR-β/STAT3 signaling	In vitro, in vivo	MIAPaCa-2, PANC-1	[149]
Glioblastoma	1 mM, 3 mM	-	Delayed cell cycle progression and potentiated the decrease of cell survival due to treatment with temozolomide	In vitro	U87MG	[150]
Oral cancer	1 mM 40 mg/kg	cyclin D1, PCNA, Bcl-2, Bax	Suppressed the invasion and migration of cancer cells through repressing ROS-activated Akt signaling Hampered vasculogenic mimicry and retarded tumorigenesis of cancer cells	In vitro, in vivo	SCC25, SCC9, Tca8113, Cal27, and FaDu	[48]
Gastric cancer	10^−4^ mol/L	Bcl-2, Bax, p53, caspase3,	Hyperbaric oxygen sensitized cancer cells to melatonin-mediated apoptosis	In vitro	SGC7901	[151]
Thyroid cancer	1, 2, 4, 8, 15 mM 25 mg/kg	caspase 3/7, PARP, cytochrome c	Reduced cell viability, inhibited cell migration and induced apoptosis Synergized with irradiation to induce cytotoxicity to thyroid cancer cells	In vitro, in vivo	8505c, ARO	[152]
Gastric cancer	1, 2, 3, 4 or 5 mM	Bax, Bcl-xL, caspase-9, caspase-3	Induced cell cycle arrest and induced apoptosis	In vitro	SGC-7901	[153]
Neural cancer	0.5, 1 mM	Bax, Bcl-2, caspase-9, cytochrome c	Mitochondrial cytochrome P450 1B1 is responsible for melatonin-induced apoptosis	In vitro	U118, SH-SY5Y, U87, U251, A172	[154]
Gastric cancer	1, 5 µM	-	Inhibited the proliferation of cancer cells by regulating the miR-16-5p-Smad3 pathway	In vitro	BSG823, SGC-7901	[155]
Head and neck squamous cell carcinoma	0.1, 0.5, or 1 mM	Bax, Bcl-2	Enhanced ROS production, increased apoptosis and mitophagy, and could be used as an adjuvant agent with rapamycin	In vitro	Cal-27, SCC-9	[94]
Ovarian cancer, colorectal cancer	0.1, 1.0, and 10 μM	-	Induced apoptosis and showed antioxidant effects	In vitro	DLD1, A2780	[156]
Cervical cancer	1 mM	JNK/Parkin/mitophagy, caspase-9	Sensitized cancer cells to cisplatin-mediated apoptosis by suppression of JNK/Parkin/mitophagy pathways	In vitro	HeLa	[100]
Melanoma Breast cancer	Melatonin: 10^−5^ − 10^−3^ M Melatonin analogues (UCM 1037): 10^−6^ − 10^−4^ M and 16 mg/Kg	Bcl-2, Bax, caspase-3	Inactivated mitophagy by suppression of JNK/Parkin, leading to the inhibition of anti-apoptotic mitophagy Sensitized cervical cancer cells to cisplatin-mediated apoptosis	In vitro, in vivo	DX3, WM-115, MCF-7, MDA-MB231	[157]
Bladder cancer	10 mg/kg 1.0 mM	caspase-3, Bcl-2, BAX	Synergized the inhibitory effects of curcumin against the growth of bladder cancer through increasing the anti-proliferation, anti-migration, and pro-apoptotic properties	In vivo, in vitro	T24, UMUC3, 5637	[158]
Colorectal cancer	1 mM	caspase-3	Increased the sensitivity of cancer cells to 5-FU	In vitro	HT-29	[159]
Lung cancer	25 mg/kg 1 mM	caspse-9, Bcl-2, PARP, cytochrome C	Increased antitumor activities of berberine through activating caspase/Cyto C and suppressing AP-2β/hTERT, NF-κB/COX-2 and Akt/ERK pathways	In vitro, in vivo	H1299, A549	[160]
Gastric cancer	1, 2 mM	caspase-3, Bcl-2, BAX	Suppressed cell viability, clone formation, cell migration and invasion and induced apoptosis	In vitro	AGS	[161]
Ovarian cancer	200 μg/100 g b.w	p53, BAX, caspase-3, Bcl-2, survivin	Promoted apoptosis	In vivo	-	[162]
Cervical cancer	1 mM	Caspase-3	Enhanced cisplatin-mediated cytotoxicity and apoptosis	In vitro	HeLa	[163]
Rhabdomyosarcoma	0.01, 0.1, 1, 2 mM	Bax, Bcl-2, caspase-3	Enhanced the sensitivity of cancer cells to apoptosis	In vitro	U57810, U23674	[164]
Hepatocellular carcinoma	2 mM	PARP, Bax	Ceramide metabolism regulated apoptotic and autophagy cell death mediated by melatonin	In vitro	HepG2	[96]
Neuroblastoma	0.25, 0.5, 1, 2 mM	-	Exerted cytotoxic potentials against cancer cells	In vitro	SH-SY5Y	[165]
Colorectal cancer	0.1–2.0 mM	HDAC4, Bcl-2, CaMKIIα	Melatonin-induced apoptosis depends on the nuclear import of HDAC4 and subsequent H3 deacetylation by CaMKIIα inactivation	In vitro	LoVo	[117]
RCC, CRC, Head and neck cancer, Prostate cancer, breast cancer	1 mM	PUMA, Mcl-1, Bcl-xL, Bim, COX-2	Enhanced antitumor effects by COX-2 downregulation and Bim up-regulation	In vitro	MDA-MB-231, Caki, HN4, HCT116, PC3	[123]
Cholangiocarcinoma	1 nM, 1 μM 0.5, 1, 2 mM	Caspase-3/7, cytochrome c	Functioned as a pro-oxidant through activating ROS-dependent DNA damage and hence leading to the apoptosis of cancer cells	In vitro	KKU-M055, KKU-M214	[166]
Lung cancer	1–5 mM	caspases-3/7	Increased cisplatin-induced cytotoxicity and apoptosis in lung cancer cells	In vitro	SK-LU-1	[167]
Gastric cancer	25, 50, 100 mg/kg	Bcl-2, Bax, p21, p53	Inhibited tumor growth by apoptosis induction	In vivo	MFC	[168]
Lung cancer	1, 5, 10 mM	caspase-3/7	Showed anticancer impacts by changing biomolecular structure of lipids, nucleic acids and proteins	In vitro	SK-LU-1	[169]
Lung cancer	10^−13^ M (subphysiological), 10^−10^ M (physiological) 10^−7^, 10^−4^, 10^− 3^ M (Cytotoxic)	CCAR2	Cell cycle and apoptosis regulator 2 (CCAR2) is critical for maintaining cell survival in the presence of melatonin	In vitro	A549, A427	[170]
Lung cancer	500 μM	Bcl-2, Bcl-xL, TRAIL	Induced apoptosis in TRAIL-resistant hypoxic tumor cells trough diminishing the anti-apoptotic signals induced by hypoxia	In vitro	A549	[114]
Breast cancer	1 nM	p53, MDM2/MDMX/p300	Enhanced p53 acetylation by regulating the MDM2/MDMX/p300 pathway	In vitro	MCF-7	[113]
Colorectal cancer	10 μM	Bax, Bcl-xL,	Activated cell death programs early and induced G1-phase arrest at the advanced phase	In vitro	HCT116	[171]
Renal cancer	0.1, 0.5,1 mM	Bim	Induced apoptosis by the upregulation of Bim expression	In vitro	Caki	[172]
Leukemia	1 mM	Bax, cytochrome c	Induced apoptosis by a caspase-dependent but ROS-independent manner	In vitro	Molt-3	[173]
Gastric cancer	10^–4^ mol/l	Caspase-3	Inhibited tumor cell proliferation and reduced the metastatic potential of cancer cells	In vitro	SGC7901	[174]
Colorectal cancer	1 mM	caspase-3/9, PARP	Potentiated the anti-proliferative and pro-apoptotic impacts of Ursolic acid in cancer cells	In vitro	SW480, LoVo	[175]
Pancreatic cancer	1.5 mmol/L 20 mg/kg	Bax, Bcl-2	Melatonin may be a pro-apoptotic and pro-necrotic molecule for cancer cells by its regulation of Bcl-2/Bax balance	In vitro, in vivo	SW-1990	[176]
Breast cancer	10^–3^ M	COX-2/PGE2, p300/NF-κB, PI3K/Akt, Apaf-1/caspase-3/9	Inhibited cell proliferation and induced apoptosis	In vitro	MDA-MB-361	[32]
Hepatocellular carcinoma	10^–9^, 10^–7^, 10^–5^, 10^–3^ μM	CHOP, Bcl-2, Bax, COX-2	Sensitized cancer cells to ER stress-mediated apoptosis by downregulating COX-2 expression, enhancing the levels of CHOP and reducing the Bcl-2/Bax ratio	In vitro	HepG2	[73]
Ovarian cancer	0, 0.5, 1, 2 mM	ERK/p90RSK/HSP27	Enhanced cisplatin-mediated apoptosis through the inactivation of ERK/p90RSK/HSP27 pathway	In vitro	SK-OV-3	[122]
Gastric cancer	2 mM	NF-κB, MAPK	Conflicting growth signals in cells may suppress melatonin efficacy in the treatment of gastric cancer	In vitro	SGC7901	[177]
Hepatocellular carcinoma	10^–3^, 10^–5^, 10^–7^, 10^–9^ mmol/L	COX-2, Bcl-2, Bax	Melatonin was shown as a novel selective ATF-6 inhibitor that can sensitize human hepatoma cells to ER stress inducing apoptosis	In vitro	HepG2	[74]
Glioma	1 μM	-	Inhibited miR-155 expression and hence repressed glioma cell proliferation, invasion and migration	In vitro	U87, U373, U251	[178]
Breast cancer	1 mM	caspase-3, hTRA, XIAP, TNFRII, P53, P21, Livin, IGF-1R, IGF-1, IGFPB-6, IGFBP-5, IGFBP-3, DR6, CYTO-C	Showed pro-apoptotic, anti-angiogenic and oncostatic properties	In vitro	MDA-MB-231, MCF-7	[179]
Leukemia	1 mM	ROS, caspase-3/8/9	Enhanced apoptotic effects of hydrogen peroxide	In vitro	HL-60	[180]
Renal cancer	1 nM	CHOP, PUMA	PUMA up-regulation contributed to the sensitizing impact of melatonin plus kahweol on apoptosis	In vitro	Caki	[181]
Pancreatic cancer	10^−8^ –10^−12^ M	Bcl-2, Bax, caspase-9	Induced pro-apoptotic pathways by interaction with the Mel-1 A/B receptors	In vitro	PANC-1	[182]
Ewing sarcoma	50 μM-1 mM	caspase-3/8/9, Bid	Showed cytoprotective effects on noncancer cells and induced apoptosis	In vitro	SK-N-MC	[183]
Glioma	1 mM	Survivin, Bcl-2	Increased cell sensitivity to TRAIL-mediated cell apoptosis	In vitro	A172, U87	[184]
Leukemia	1 mM 250 mg/kg	Bax, Bcl-2, p53	Enhanced radiation-mediated apoptosis in cancer cells, while decreasin radiation-meditated apoptosis in normal cells	In vitro, in vivo	Jurkat	[185]
Breast cancer	1 nM	Caspase-7/9, p53, MDM2, PARP, Bcl-2, Bax	Induced apoptosis in cancer cells	In vitro	MCF-7	[116]
Hepatocellular carcinoma	1000–10,000 μM	caspase-3/8/9, PARP, cytochrome c, Bax, p53, p21	Induced cell cycle arrest and apoptosis	In vitro	HepG2	[115]
Pheochromocytoma	100 μM	GSH	Apoptotic and antioxidant effects	In vitro	PC12	[186]
Neuroblastoma	100 μM	Caspase-3	Induced apoptosis	In vitro	SK-N-MC	[187]
Leukemia Cervical cancer	50 μM	Caspase-3	Protectted normal and cancer cells against genotoxic treatment and apoptosis induced by idarubicin	In vitro	K562, HeLa	[188]
Colorectal cancer	1 mM	Caspase-3	Potentiated flavone-mediated apoptosis in cancer cells	In vitro	HT-29	[189]
Breast cancer	1 nM	Bax, p53, p21, WAF1, bcl-XL, bcl-2	Decreased cancer cell proliferation through regulating cell-cycle length by the control of the p53-p21 pathway	In vitro	MCF-7	[190]
Esophageal cancer	0–5 mM 25 mg/kg	PARP, caspase-3/7/8	Increased cytotoxicity of 5-Fu	In vivo, in vitro	KYSE30, KYSE150, KYSE410, KYSE520	[191]

Melatonin induces dephosphorylation and nuclear import of histone deacetylase 4 (HDAC4) in cancer cells; melatonin exerts this effect through inactivation of Ca^2+^/calmodulin-dependent protein kinase II alpha (CaMKIIα), leading to the H3 acetylation on Bcl-2 promoter and subsequent reduction of Bcl-2 expression [117]. Furthermore, inhibition of HDAC9 expression is a mechanism of melatonin to promote apoptosis in non-small cell lung cancer; the increased level of HDAC9 in patients with non-small cell lung cancer is correlated with worse overall survival and poor prognosis [118]. Melatonin promotes TNF-α-mediated apoptosis via inhibiting mitophagy in tumor cells. Since activation of mitophagy suppresses mitochondrial apoptosis, inhibition of mitophagy by melatonin results in the repression of mitochondrial potential, elevation of ROS generation, augmentation of mPTP opening rate and up-regulation of cytochrome c expression and caspases activity. Melatonin inhibits autophagy in tumor cells through inhibiting CaMKII activity leading to the suppression of Parkin expression [119]. In diethylnitrosamine (DEN)-induced hepatocellular carcinoma (HCC), melatonin increases therapeutic potential of mesenchymal stem cells (MSCs) through reduction of oxidative stress and inflammation, and induction of apoptosis [120].

Melatonin has been reported to increase therapeutic potential of anti-cancer agents, which this effect may result from its stimulatory effect on apoptosis. Co-treatment of melatonin and pterostilbene in colorectal cancer cells synergically enhances ROS production and apoptosis. Combination of these two agents upregulates the mRNA level of miR-25-5p, which this results in the activation of PARP and sex-determining region Y-Box10 (SOX10), and attenuation of Bcl-xL, neural precursor cell expressed developmentally downregulated protein 9 (NEDD9), and SOX9 expressions [121]. Melatonin synergically enhances anticancer potential of cisplatin through inducing apoptosis; melatonin increases the effect of cisplatin to the inhibition of ERK phosphorylation and induction of 90-kDa ribosomal S6 kinase (p90RSK) and heat shock protein 27 (HSP27) dephosphorylation [122]. Treatment with melatonin enhances ER stress–mediated apoptosis in tunicamycin-treated cancer cells; this effect is associated with the down-regulation of COX-2 and Bcl-2 expressions and up-regulation of Bim, CHOP and Bax expressions [73]. Melatonin inhibits tunicamycin-induced COX-2 activation in tumor cells through inhibiting NF-ĸB and p38 MAPK activation and p65 nuclear translocation [123]. Combination of melatonin with phenylarsine oxide also induces endoplasmic reticulum stress-induced cell death, accompanied by JNK activation, PARP cleavage, ROS generation and caspase-3 activation [124].

## Conclusions

This review summarizes the anti-carcinogenic potentials of melatonin by evaluating various signaling pathways. Melatonin inhibits proliferation of cancer cells through triggering cell cycle arrest and causes cell death by induction of apoptosis. Melatonin suppresses metastasis angiogenesis, and proliferation of cancer cells through affecting various signaling pathways in tumor cells. Melatonin also regulates autophagy pathway in cancer cell by affecting oxidative stress condition in tumor cells. These findings suggest that melatonin may increase the sensitivity of cancer cells to anti-cancer agents and may be a potential treatment for cancers either alone or in combination with other anti-cancer drugs. However, further clinical studies are needed to clarify the effect of this molecule in different cancers and obtain affective dose of melatonin for patients with cancer.

## Data Availability

Not applicable.

## Abbreviations

JNK: C-Jun N-terminal kinase
ROS: Reactive oxygen species
VEGF: Vascular endothelial growth factor
TNF-α: Tumor necrosis factor-α
IL-2: Interleukin-2
Nrf2: Nuclear factor erythroid 2-related factor 2
Apaf-1: Apoptotic protease activating factor-1
COX-2: Cyclooxygenase-2
Sirt1: Sirtuin
HIF: Hypoxia-inducible factor
TRAIL: TNF-related apoptosis-inducing ligand
PARP-1: Poly [ADP-ribose] polymerase 1
ATG: Autophagy related genes
IRE1: Inositol-requiring enzyme 1
ATF6: Activating transcription factor 6
ERK: Extracellular signal-regulated kinase
MEK: Mitogen-activated protein kinase kinase
MAPK: Mitogen-activated protein kinase
CHOP: CCAAT-enhancer-binding proteins homologous protein
PUMA: P53-upregulated modulator of apoptosis
Bcl-2: B cell lymphoma-2
Bim: Bcl-2-interacting mediator of cell death
PI3K: Phosphatidylinositol-3-kinase
Akt: Protein kinase B
UPR: Unfolded protein response
ATF6α: Activating transcription factor 6α
ER: Endoplasmic reticulum
PERK: Protein kinase RNA-like ER kinase
ERK: Extracellular signal-regulated kinase
SPHK1: Sphingosine kinase 1
IFN-γ: Interferon-gamma
PGC1A: Peroxisome proliferator-activated receptor gamma coactivator 1A
NQO-1: NAD(P)H:quinone oxidoreductase
HO-1: Heme oxygenase-1
FADD: Fas-associated death domain
tBid: Truncated Bid
ASK1: Apoptosis signal-regulating kinase 1
TRAF2: IRE1α-TNF receptor-associated factor 2
ERKs: Extracellular-regulated protein kinases
CaMKIIα: Ca^2^^+^/calmodulin-dependent protein kinase II alpha
DEN: Diethylnitrosamine
HCC: Hepatocellular carcinoma
MSCs: Mesenchymal stem cells
NEDD9: Neural precursor cell expressed developmentally downregulated protein 9
p90RSK: 90-KDa ribosomal S6 kinase
HSP27: Heat shock protein 27
